# Supplementary material for: Integrating nutrition and physical activity promotion: A scoping review
Source: PLoS One. 2020 Jun 5;15(6):e0233908. doi: 10.1371/journal.pone.0233908 (PMC7274388; doi:10.1371/journal.pone.0233908)
Supplement: S1 Table — Adapted from WHO (2011) ‘Good Practice Appraisal Tool for Obesity Prevention Programmes, Projects, Initiatives and Interventions’. Studies marked with * are to be considered in conjunction with other studies on the same interventions. (PDF) [file pone.0233908.s001.pdf]

SUPPLEMENTARY TABLE 1: Critical Appraisal

Y = Yes<br>N = No<br>? = unclear<br>- = not applicable	Bock (2014)	Bolton (2017)	Cheadle (2018)	Colchamiro (2010)	Correa (2010)	Cradock (2016)	Davis (2013)	FF/IDS (2017)*	Folta(2015)	Griffin (2018)	Hinkle (2018)	Irwin (2012)	Jung (2018)	Lang (2017)	Linton (2014)	Lyn (2013)	Madsen (2015)	MCA-Mongolia	McAuley (2010)	McDavid (2016)	McIsaac(2017)	Meinen (2018)	Miller (2018)	Mukhina (2014)	Paes-Sousa (2014)*	Patriarca (2016)*	Payne (2018)	Perez-Escamilla	Reeve (2015)	RIDOH (2018)*	Rito (2013)	Sekhobo (2012)	Soler (2014)	SPRING (2014)	TYF (2014)	Weber (2017)
Clear definition of study population	Y	Y	Y	Y	Y	Y	Y	Y	Y	Y	Y	Y	Y	Y	Y	Y	Y	Y	Y	Y	Y	N	Y	Y	Y	Y	Y	Y	N	Y	Y	Y	Y	Y	N	Y
Clear definition of outcomes and outcome assessment	Y	Y	Y	N	Y	Y	N	N	Y	Y	Y	Y	Y	Y	Y	Y	Y	N	Y	Y	Y	N	Y	Y	N	N	Y	N	N	N	Y	N	Y	N	N	Y
Independent assessment of outcome parameters	Y	Y	Y	Y	N	Y	Y	-	Y	Y	Y	Y	Y	Y	Y	Y	Y	-	Y	Y	Y	N	Y	Y	-	-	Y	-	-	-	Y	-	Y	Y	-	Y
Sufficient duration of follow-up (at least 6–12 months)	Y	Y	Y	N	N	Y	N	Y	N	N	-	Y	Y	Y	Y	Y	Y	Y	Y	Y	N	-	Y	-	-	-	Y	-	-	Y	Y	-	Y	Y	-	Y
Aims of intervention are clearly described	Y	N	Y	Y	Y	Y	Y	Y	Y	Y	Y	Y	Y	Y	Y	Y	Y	Y	Y	Y	Y	Y	Y	Y	Y	Y	Y	Y	Y	Y	Y	Y	Y	Y	Y	Y
Intervention combines healthy eating and physical activity	Y	Y	Y	Y	Y	Y	Y	Y	Y	Y	Y	Y	Y	Y	Y	Y	Y	Y	Y	Y	Y	Y	Y	Y	Y	Y	Y	Y	Y	Y	Y	Y	Y	Y	Y	Y
SMART objectives are clearly described	Y	Y	Y	N	Y	Y	N	-	Y	Y	N	Y	Y	Y	Y	Y	Y	N	Y	Y	Y	N	Y	Y	-	Y	Y	-	-	Y	Y	Y	-	-	-	Y
Based on current scientific knowledge / theoretical models / previous experience	Y	Y	Y	Y	Y	Y	Y	Y	Y	Y	?	Y	Y	Y	Y	Y	Y	Y	Y	Y	Y	Y	Y	Y	Y	Y	Y	Y	Y	Y	Y	Y	Y	Y	Y	Y
Acts in coherence with existing diet / physical activity guidelines	Y	?	Y	Y	Y	Y	Y	Y	Y	Y	?	Y	Y	Y	Y	Y	Y	?	Y	Y	?	?	Y	Y	Y	Y	Y	Y	Y	Y	Y	Y	Y	Y	Y	Y
A needs assessment has been performed	?	Y	Y	Y	Y	?	Y	Y	N	?	?	?	Y	Y	Y	?	?	Y	?	Y	?	?	Y	?	Y	Y	Y	?	?	Y	?	Y	Y	Y	Y	?
Planned key activities are relevant to the needs of the target group	Y	Y	Y	Y	Y	Y	Y	Y	Y	Y	Y	Y	Y	Y	Y	Y	Y	Y	Y	Y	Y	Y	Y	Y	Y	Y	Y	Y	Y	Y	Y	Y	Y	Y	Y	Y
The activities also address environmental factors	Y	Y	Y	Y	Y	N	Y	Y	Y	Y	Y	Y	Y	Y	Y	Y	Y	Y	Y	Y	Y	Y	Y	Y	Y	Y	Y	Y	Y	Y	Y	Y	Y	Y	Y	Y
The intervention is designed to have lasting effect on the risk factors	?	Y	Y	?	Y	?	Y	Y	Y	?	Y	?	Y	Y	Y	Y	Y	Y	Y	Y	Y	?	Y	Y	Y	Y	Y	Y	Y	Y	Y	Y	Y	Y	Y	Y

The activities take place within structures that can carry on the intervention	Y	Y	Y	Y	?	Y	Y	Y	Y	?	Y	Y	Y	Y	Y	Y	Y	Y	Y	Y	Y	Y	Y	Y	Y	Y	Y	Y	Y	Y	Y	Y	Y	Y	Y	Y
The target group(s) is/are clearly stated	Y	Y	Y	Y	Y	Y	Y	Y	Y	Y	Y	Y	Y	Y	Y	Y	Y	Y	Y	Y	Y	Y	Y	Y	Y	Y	Y	Y	Y	Y	Y	Y	Y	Y	Y	Y
There is a special focus on vulnerable groups	Y	N	Y	Y	Y	N	Y	Y	N	Y	N	Y	Y	N	Y	Y	Y	Y	Y	Y	Y	Y	Y	Y	Y	Y	N	Y	Y	Y	Y	Y	Y	Y	Y	Y
The intervention aims to empower target group(s)	Y	Y	Y	Y	Y	N	Y	Y	Y	Y	Y	Y	Y	Y	Y	Y	Y	Y	Y	Y	Y	Y	Y	Y	Y	Y	Y	Y	Y	Y	Y	Y	Y	Y	Y	Y
The target group(s) has/have been involved in setting objectives and designing intervention	N	N	?	Y	Y	N	Y	Y	Y	N	Y	N	Y	Y	Y	Y	N	Y	N	N	N	N	N	N	Y	Y	Y	N	?	Y	N	N	?	?	N	N
The intervention involves professionals from different sectors	Y	Y	Y	Y	Y	Y	Y	Y	Y	Y	Y	Y	Y	Y	Y	Y	Y	Y	Y	Y	Y	Y	Y	Y	Y	Y	Y	Y	Y	Y	Y	Y	Y	Y	Y	Y
Demographic and socioeconomic factors of the target population are measured	Y	Y	Y	N	N	Y	N	Y	N	Y	N	Y	Y	Y	Y	N	Y	N	Y	N	Y	N	Y	N	Y	-	Y	N	N	-	Y	N	-	-	-	Y
The statistical methods are described	Y	Y	Y	N	N	Y	N	Y	N	Y	N	Y	Y	Y	Y	Y	Y	N	Y	Y	Y	N	Y	N	Y	-	Y	-	N	-	Y	Y	-	-	-	Y
External and/or internal evaluations have been performed	Y	Y	Y	Y	Y	Y	Y	Y	Y	Y	Y	Y	Y	Y	Y	Y	Y	Y	Y	Y	Y	Y	Y	Y	Y	Y	Y	Y	Y	Y	Y	Y	Y	Y	Y	Y
Total Y per study	19	18	21	16	17	16	17	19	17	17	14	19	22	21	22	20	20	16	20	20	18	11	21	17	18	17	21	14	13	18	20	17	18	17	14	20
Total N per study	1	3	0	5	4	4	5	1	5	2	4	1	0	1	0	1	1	4	1	2	2	7	1	3	1	1	1	3	4	1	1	3	0	1	3	1
Total ? per study	2	1	1	1	1	2	0	0	0	3	3	2	0	0	0	1	1	1	1	0	2	3	0	1	0	0	0	1	2	0	1	0	1	1	5	1
Total n/a per study	0	0	0	0	0	0	0	2	0	0	1	0	0	0	0	0	0	1	0	0	0	1	0	1	3	4	0	4	3	3	0	2	3	3	0	0
