# Supplementary material for: Integrating nutrition and physical activity promotion: A scoping review
Source: PLoS One. 2020 Jun 5;15(6):e0233908. doi: 10.1371/journal.pone.0233908 (PMC7274388; doi:10.1371/journal.pone.0233908)
Supplement: S2 Table — Legend: Y = Yes; N = No; N/A = unclear / lack of detail for in-depth analysis of single components; - = need for in-depth analysis of single components. (PDF) [file pone.0233908.s002.pdf]

SUPPLEMENTARY TABLE 2: Assessment of double duty action eligibility

	First author / year	Platform	Programme	Setting	Awareness / focus on demographic status vulnerability	Awareness / focus on socioeconomic status vulnerability	DD-potential based on conducive environment focus	Consistent with DD 'do no harm' principle
1	Bock (2014)	Health service Family focus	The Health Initiative Program for Kids (HIP Kids)	Canada	Y	N	Y	N/A
2	Bolton (2017)	Community	The Health-Promoting Communities: Being Active Eating Well initiative	Australia	Y	?	Y	N/A
3	Cheadle (2018)	Community	Kaiser Permanente's Healthy Eating Active Living Zones Initiative	USA	Y	Y	Y	N/A
4	Colchamiro (2010)	Early care	Massachusetts Women, Infants, and Children (WIC) Program	USA	Y	N	Y	N/A
5	Correa (2010)	Community	CAN DO Houston	USA	Y	Y	Y	N/A
6	Cradock (2016)	OST (out-of-school time)	The Out of School Nutrition and Physical Activity (OSNAP) Initiative	USA	Y	Y	Y	N/A
7	Davis (2013)	Preschool	CHILE: preschool intervention for obesity prevention in Head Start	USA	Y	Y	Y	N/A
8	Folta (2015)	OST	Volunteer-Led Out-of-School Time Programs	USA	Y	Y	Y	N/A
9	Griffin (2018)	Higher risk group	My Quest, using text messaging to improve behaviours among low-income women	USA	Y	Y	Y	N/A
10	Hinkle (2018)	Community	Food & Fitness (F&F) Community Partnerships	USA	Y	Y	Y	N/A
11	Irwin (2012)	Community-School-home	Get Fit with the Grizzlies initiative	USA	Y	N	Y	N/A
12	Jung (2018)	Community Family focus	Healthy Together (HT) physical activity and healthy eating family program	Canada	Y	Y	Y	N/A
13	Lang (2017)	Workplace	The National Healthy Worksite Program	USA	N	N	Y	N/A
14	Linton (2014)	Community	The San Diego County Childhood Obesity Initiative (COI)	USA	Y	Y	Y	N/A
15	Lyn (2013)	Child care centre	Wellness policy and training program in 24 child care centres in Georgia	USA	Y	Y	Y	N/A
16	Madsen (2015)	School	The Healthy Schools Program	USA	Y	N	Y	N/A
17	MCA-Mongolia	Community	Millennium Challenge Account – Mongolia 5-year NCD prevention programmes	Mongolia	Y	Y	Y	N/A

	(2013)							
18	McAuley (2010)	Community	The APPLE (A Pilot Program for Lifestyle and Exercise) project	New Zealand	Y	N	Y	N/A
19	McDavid (2016)	Child care centre	Growing Fit: engaging early care environments in preventing obesity	USA	Y	Y	Y	N/A
20	McIsaac (2017)	School	Health-Promoting Schools program in Nova Scotia, Canada	Canada	Y	Y	Y	N/A
21	Meinen (2018)	Child care centre	The state-wide Wisconsin Early Childhood Obesity Prevention Initiative	USA	Y	N	Y	N/A
22	Miller (2018)	School	Let's Move! Active Schools (LMAS)	USA	Y	N	Y	N/A
23	Mukhina (2014)	School	BeHealthy Charities Aid Foundation Program	Russia	Y	Y	Y	N/A
24	Paes-Sousa/FF-IDS (2014-2017)	Community	Zero Hunger/Programa Academia Saúde: Brazil’s food, nutrition and health promotion governance plan	Brazil	Y	Y	Y	-
25	Patriarca/ RIDOH (2016-2018)	Community	Rhode Island’s Health Equity Zones	USA	Y	Y	Y	N/A
26	Payne (2018)	Workplace	Workplace culture of health (COH)	USA	N	N	Y	N/A
27	Perez-Escamilla (2018)	School	Mondelez International Foundation Healthy Lifestyles Programs	China, India, Brazil, South Africa, UK, Germany, Mexico	Y	Y	Y	N/A
28	Reeve (2015)	Community	State and Municipal Innovations in Obesity Policy	USA	Y	Y	Y	N/A
29	Rito (2013)	Community	Program Obesity Zero (POZ), targeting children from five municipalities	Portugal	Y	Y	Y	N/A
30	Sekhobo (2012)	Preschool	New York State childhood obesity prevention program for Women, Infants and Children (NY Fit WIC)	USA	Y	Y	Y	N/A
31	Soler (2014)	Community	The CDC’s Communities Putting Prevention to Work (CPPW) initiative	USA	Y	Y	Y	N/A
32	SPRING (2014)	Community	Prospera/EsIAN: Mexico’s National Integrated Nutritional Strategy	Mexico	Y	Y	Y	-
33	TYF (2014)	Community	The Health Innovation Network and Young Foundation Obesity South London Strategy	UK	Y	Y	Y	N/A
34	Weber (2017)	School	Promoting Physical Activity and Balanced Diets in school with high proportion of children with a migration background	USA	Y	Y	Y	N/A
