# Supplementary material for: Integrating nutrition and physical activity promotion: A scoping review
Source: PLoS One. 2020 Jun 5;15(6):e0233908. doi: 10.1371/journal.pone.0233908 (PMC7274388; doi:10.1371/journal.pone.0233908)
Supplement: S1 Text — (DOCX) [file pone.0233908.s003.docx]

**S3 Supplementary information**

Consistently with the Cochrane PROGRESS-Plus framework, we applied an equity lens to the classification of studies. The acronym PROGRESS includes the following factors: place of residence, race/ethnicity/culture/language, occupation, gender/sex, religion, education, socioeconomic status, and social capital. The PLUS addition includes: a) personal characteristics associated with discrimination (e.g. age, disability); b) features of relationships (e.g. smoking parents, excluded from school); and c) time-dependent relationships (e.g. leaving the hospital, respite care, other instances where a person may be temporarily at a disadvantage).

Whilst most interventions and initiatives reviewed were not originally framed as double duty actions, and the information available did not allow for a detailed analysis of inputs and outputs that may be classified as fulfilling double duty, we identified some of the elements that justify their inclusion as potential DDA. Demographic and socioeconomic factors associated with malnutrition were taken as important markers of whether interventions hold potential for double duty. These elements encompass multiple vulnerabilities determined by underlying and structural determinants of malnutrition, as well as recognised environmental exposures to risk throughout the lifecycle.

**Demographic status vulnerability**:

Interventions were classified based on their recorded awareness of/focus on relevant demographic factors. These included considerations on age and gender - e.g. the importance of targeting infants and children; the role of adolescent girls, who will become mothers of the next generations of children, with implications for nutrition, child development, nutrition habit formation and health; as well as attention paid to groups considered at higher risk of overweight/obesity in the literature. Demographic characteristics may reinforce inequalities between individuals, households and communities. The type of occupations available to some male and female adults from low-income backgrounds reflect underlying and structural causes of gendered differences. Such correlations may be dependent on educational status, and the likelihood to work in labour-intensive industries with low pay, as opposed to having access to work in higher paid positions, or in environments where corporate-wellness programmes (including nutrition, physical activity and health monitoring) are being promoted. Other factors, such as marital status, religion and culture, average age at marriage, average size of families in different communities, and so on, are likely to overlap with socioeconomic differences, resulting in mutually reinforcing inequalities.

**Socioeconomic status vulnerability**:

Classification of interventions and initiatives as being potential DDAs was also based on recorded awareness of/focus on the link between nutrition/activity/health and socioeconomic position of individuals, households and communities in given settings. Factors that influence circumstances and behaviour in low income settings include availability and access to food, prioritisation of price over nutritional properties, impact of number of children in the household on quantity and quality of food available to each member, cash availability at different times of the year/month, seasonal variations in food access, provision of less/lower-quality food at home when children have access to cooked food in schools, holiday hunger when children from poor households have no access to the quantity and quality of food being provided at school, and so on. Known links between socioeconomic position, circumstances and behaviours adopted by individuals and households to cope with limitations, make these factors relevant for assessing whether interventions carry potential for double duty action. For these reasons, interventions and initiatives that showed an awareness of and/or focus on differences in endowments of individuals and groups, and consequent inequalities in health outcomes and life expectancy within and across communities, were considered to have potential for double duty.

**Conducive environment**:

The consideration given to mutually reinforcing differences and inequalities and their environmental determinants in the studies examined is likely to be important for all forms of malnutrition. The level of detail provided is not sufficient for carrying out a more methodical analysis and make more definite statements on their eligibility as actions that explicitly fulfil double duty. Nevertheless, they do act on underlying and structural determinants. A stress on environmental factors has potential for impacting on multiple drivers and facilitate broader changes conducive to double duty. This provides opportunities for better quality of life - not only for obesity control and prevention, but also for undernutrition - for those individuals, families and communities that may be exposed to risk as a consequence of low income and other vulnerabilities.

**Do-no-harm principle**:

There is often an indication that interventions are based on current scientific knowledge/theoretical models/previous experience, or act in coherence with existing diet/physical activity guidelines (see S1 Table). However, we cannot directly verify this. Even when sectoral guidelines and policy advice are mentioned, details of the exact inputs and how these may impact on other forms of malnutrition are not specified. While this constitutes a limitation for our review, it highlights a gap in the literature. The need to include a more conscious analysis of how individual components may impact on other forms of malnutrition is important for future design, implementation and evaluation. Because we are unable to conduct this analysis (see S2 Table), the column based on consistency of interventions with the ‘do no harm’ principle may seem superfluous. Yet, it stresses the fact that the information needed for the type of analysis that will advance the study and application of DDA, is not there yet. Furthermore, without this knowledge base, it is difficult to understand whether and how ‘retrofitting’ for DDA can be applied to existing interventions that tackle single forms of malnutrition.
